# Antibacterial therapy in central nervous system infections – breakpoints versus minimal inhibitory concentrations related to body fluid levels

**DOI:** 10.1007/s15010-025-02678-7

**Published:** 2025-11-07

**Authors:** Roland Nau, Jana Seele, Utz Reichard, Fritz Sörgel

**Affiliations:** 1 Institute of Neuropathology, University Medical Center, Göttingen, Germany; 2 Department of Geriatrics, Protestant Hospital Göttingen-Weende, An der Lutter 24, 37075 Göttingen, Germany; 3 Institute for Biomedical and Pharmaceutical Research (IBMP), Nürnberg- Heroldsberg, Germany; 4 Amedes MVZ for Laboratory Medicine, Medical Microbiology and Infectiology, Göttingen, Germany; 5 Institute of Pharmacology, West German Heart and Vascular Center, University of Duisburg-Essen, Essen, Germany

**Keywords:** Minimum inhibitory concentration (MIC), Minimum bactericidal concentration (MBC), Breakpoint, Antibiotics, Cerebrospinal fluid, Meningitis, Ventriculitis, Pharmacokinetics, Pharmacodynamics

## Abstract

Breakpoints to define the susceptibility of pathogens to antibiotics are becoming increasingly popular as guidance for antimicrobial therapy. Some breakpoints consider divergent concentrations of antibiotics in different compartments, others list one breakpoint for infections at all sites. Compared to the determination of exact minimal inhibitory concentrations (MICs) and relation of these MICs to concentrations achievable in ventricular CSF, the exclusive use of breakpoints for central nervous system (CNS) infections is a setback. The lack of the determination of exact MICs may be a risk, particularly when no clinical breakpoints for CNS infections have been defined. Moreover, the current practice of MIC determination of β-lactam/β-lactamase inhibitor combinations with fixed β-lactamase inhibitor concentrations ignores that often these β-lactamase inhibitor concentrations are not attained in CSF with established antibiotic regimens. In CNS infections, we strongly recommend the exact determination of MICs and their interpretation in relation to the true antibiotic concentrations in the infected compartment.

## Introduction

As a consequence of the barriers surrounding the central nervous system (CNS) [1], bacterial CNS infections are a therapeutic challenge. Whereas the incidence of community-acquired meningitis is decreasing mainly because vaccination programs, the incidence of nosocomial meningitis, ventriculitis and intracranial abscesses is rising [2, 3]. In most countries, nosocomial CNS infections frequently are caused by pathogens with a reduced susceptibility to standard antibiotics. Some nosocomial pathogens, in particular *Acinetobacter* and *Klebsiella* spp. strains, are resistant to most antibiotics established for the treatment of CNS infections. CNS infections with Gram-negative bacteria resistant to all traditional β-lactam antibiotics and fluoroquinolones [known as difficult-to-treat resistance (DTR)] pose particular problems, which often require the use of newer β-lactam/ β-lactamase combinations [4].

Classically, susceptibility of a pathogen to antibiotics is assessed by the measurement of the minimal inhibitory concentration (MIC) by macro- or microdilution methods or (less accurate) by disk diffusion or E-test. The more resource-consuming minimal bactericidal concentration (MBC) is determined at times, when a large difference between MIC and MBC is suspected. As a consequence of commercialization and shortage of manpower in many health services, several (semi)automatic laboratory analyzers have been introduced into clinical routine, which do not determine MICs or MBCs, but classify bacteria as “susceptible (standard dosing regimen)” (S), “susceptible (increased exposure; high likelihood of therapeutic success when exposure to the agent is increased by adjusting the dosing regimen or by its concentration at the site of infection)” (I) or “resistant” (R) [5, 6]. These “breakpoints are valid also for special situations such as endocarditis and meningitis”, “unless otherwise stated” [6].

In this comment on the hazards of guiding antibiotic therapy by breakpoints as a replacement for exact MICs or MBCs in relation to the antibiotic concentrations in the infected compartment, meningitis and ventriculitis may serve as proxies for other infections in deep compartments. Unlike infections in other deep compartments (e.g. endocarditis, intraabdominal abscesses), samples from different regions of the CSF compartment can be used for the measurement of antibiotic concentrations, which resemble, but often are not identical to the antibiotic concentrations at the site of infection [1, 7] making therapeutic drug monitoring in CNS infections meaningful.

## Pathophysiology

The CNS is surrounded by the blood-brain and blood-CSF barrier, which impede the entry of antibiotics, and simplified can be considered a lipid bilayer. In healthy individuals, leaky regions comprise approximately 1:5,000 of the entire capillary surface area [1]. Leaks become more abundant during CNS inflammation. After entry into the CNS, antibiotics are removed by (a) the CSF bulk flow (approx. 500 ml/24 h in health, often decreasing as a consequence of the obstruction of outflow pathways during CNS inflammation), (b) retrograde diffusion across the barriers and (c) several outward transport systems with affinity to antibiotics and (d) the glymphatic system [1, 7, 8]. As a consequence of the CSF bulk flow from the ventricles to the basal cisterns, then to the convexities and to the spinal canal, during systemic treatment lumbar concentrations of compounds entering the CNS from the bloodstream usually are higher than ventricular levels [1, 7]. In individual patients, concentrations in CSF or the extra- or intracellular fluid (ECF/ICF) of the infected tissue are hard to predict stressing the necessity of drug monitoring in DTR infections. Antibiotic CSF concentrations do not equal the concentrations in brain or medulla ECF, however, they provide the closest estimates available in clinical routine, unless the critically ill patient is monitored by cerebral microdialysis [9].

## Pharmacokinetics of antibiotics in the CNS

Ventricular drug concentrations measured in patients in the absence of meningeal inflammation represent the minimum concentrations which can be encountered during systemic antibiotic therapy in CNS infection, particularly at the beginning or during resolution of inflammation [7]. With the exception of severe meningitis or lumbar CSF during a spinal CSF blockade, protein binding in CSF is low because of the low CSF protein content. As a simplification we assume that the area of the concentration-time curve of the unbound fraction of the antibiotic in CSF (AUC_CSF free_) approximately equals the concentration-time curve of the antibiotic in CSF (AUC_CSF_) and use AUC_CSF free_ and AUC_CSF_ as equivalents. During intravenous or oral therapy, lumbar CSF concentrations are usually higher than ventricular CSF concentrations [7]. For moderately lipophilic drugs (e.g. linezolid, metronidazole, levofloxacin), concentration-time curves in serum and CSF after the initial distribution phase roughly run in parallel, and ratios of the areas under the concentration-time curves in CSF and of the unbound fraction in serum (AUC_CSF_/AUC_S free_) approximate 1. For hydrophilic drugs (e.g. almost all β-lactam antibiotics, glycopeptides, aminoglycosides), AUC_CSF_/AUC_S free_ are far below 1 (often < 0.2), and the concentration-time curves in CSF lag behind those in serum. No antibiotic administered intravenously reaches an AUC_CSF_/AUC_S free_ >1 [7]. As the concept of the sink action of the CSF [1] suggests, free concentrations of antimicrobial agents in the ECF tend to be higher than those in the adjacent CSF [10, 11].

High antibiotic concentrations in the CSF and cerebral ECF can be achieved after intraventricular drug administration. Injection into the lumbar CSF does not reliably achieve therapeutic concentrations in all parts of the CSF compartment and the adjacent ECF [12, 13]. Breakpoints (Table 1) do not consider CSF concentrations achieved after intraventricular drug injection [13].

**Table 1:** Systemic versus CNS infection breakpoints of typical pathogens causing community-acquired and nosocomial meningitis and ventriculitis [5, 6]

Microorganism, antibiotic	Breakpoint systemic infection EUCAST/CLSI		Breakpoint CNS infection EUCAST/CLSI	
	S	R EUCAST: > CLSI: ≥	S	R EUCAST: > CLSI: ≥
***Streptococcus pneumoniae***				
**Benzylpenicillin**	**≤0.06/ ≤2**	**>1/ ≥8**	**≤0.06/ ≤0.06**	**>0.06/ ≥0.12**
**Ampicillin, amoxicillin**	**≤0.5/≤ 2**	**>1/ ≥8**	**≤0.5/#**	**>0.5/#**
**Cefotaxime, ceftriaxone**	**≤0.5/ ≤1**	**>2/ ≥4**	**≤0.5/ ≤0.5**	**>0.5/ ≥2**
**Cefepime**	**≤1/ ≤1**	**>2/ ≥4**	**≤1/≤0.5**	**>2/ ≥2**
**Meropenem**	**≤2/** ≤0.25	**>2/** ≥1	≤ **0.25/** ≤0.25	**>0.25/** ≥1
Vancomycin	≤ 2/ ≤1	>2/ ≥1	≤ 2/ ≤1	>2/ ≥1
Levofloxacin	≤ 0.001/≤ 2	>2/ ≥8	≤ 0.001/ ≤2	>2/ ≥8
Moxifloxacin	≤ 0.5/ ≤1	>0.5/ ≥4	≤ 0.5/ ≤1	>0.5/ ≥4
Rifampicin	≤ 0.125/ ≤1	>0.125/ ≥4	≤ 0.125/ ≤1	>0.125/ ≥4
***Neisseria meningitidis***				
Benzylpenicillin	≤ 0.25/ ≤0.06	>0.25/ ≥0.5	≤ 0.25/ ≤0.06	>0.25/ ≥0.5
Ampicillin, amoxicillin	≤ 0.125/≤0.12	>1/ ≥2	IE/ ≤0.12	IE/ ≥2
Cefotaxime, ceftriaxone	≤ 0.125/ ≤0.12	>0.125/--	≤ 0.125/ ≤0.12	>0.125/--
Meropenem	≤ 0.25/ ≤0.25	>0.25/--	≤ 0.25/ ≤0.25	>0.25/--
Ciprofloxacin	≤ 0.016/ ≤0.03	>0.016/ ≥0.12	≤ 0.016/ ≤0.03	>0.016/ ≥0.12
***Haemophilus influenzae***				
Ampicillin, amoxicillin	≤ 1/ ≤1	>1/ ≥4	IE/ ≤1	IE/ ≥4
Cefotaxime, ceftriaxone	≤ 0.125/ ≤2	>0.125/--	≤ 0.125/ ≤2	>0.125/--
**Meropenem**	**≤2/** ≤0.5	**>2/**--	**≤0.25/** ≤0.5	**>0.25/**--
Ciprofloxacin	≤ 0.03/ ≤1	>0.03/--	≤ 0.03/ ≤1	>0.03/--
Levofloxacin	≤ 0.06/ ≤2	>0.06/--	≤ 0.06/ ≤2	>0.06/--
Moxifloxacin	≤ 0.125/ ≤1	>0.125/--	≤ 0.125/ ≤1	>0.125/--
***Listeria monocytogenes***				
Ampicillin	≤ 1/ ≤2	>1/ --	≤ 1/ ≤2	>1/ --
Meropenem	≤ 0.25/ ≤0.25	>0.25/ --	≤ 0.25/ ≤0.25	>0.25/ --
Trimethoprim-sulfamethoxazole	≤ 0.06/ ≤0.5^§^	>0.06/ --^§^	≤ 0.06/ ≤0.5^§^	>0.06/ --^§^
***Staphylococcus aureus***				
Benzylpenicillin	≤ 0.125/ ≤0.125	>0.125/ ≥0.25	≤ 0.125/ ≤0.125	>0.125/ ≥0.25
Oxacillin	--/ ≤2	>2/ ≥4	--/ ≤2	>2/ ≥4
Ciprofloxacin	≤ 0.001/ ≤1	>2/ ≥4	≤ 0.001/ ≤1	>2/ ≥4
Levofloxacin	≤ 0.001/ ≤1	>1/ ≥4	≤ 0.001/ ≤1	>1/ ≥4
Moxifloxacin	≤ 0.25/ ≤0.5	>0.25/ ≥2	≤ 0.25/ ≤0.5	>0.25/ ≥2
Vancomycin	≤ 2/ ≤2	>2/ ≥16	≤ 2/ ≤2	>2/ ≥16
Linezolid	≤ 4/ ≤4	>4/ ≥8	≤ 4/ ≤4	>4/ ≥8
Rifampicin	≤ 0.06/ ≤1	>0.06/ ≥4	≤ 0.06/ ≤1	>0.06/ ≥4
***Coagulase-negative staphylococci***				
Oxacillin	--/ ≤0.5	>0.25/ ≥1	--/ ≤0.5	>0.25/ ≥1
Ciprofloxacin	≤ 0.001/ ≤1	>2/ ≥4	≤ 0.001/ ≤1	>2/ ≥4
Levofloxacin	≤ 0.001/ ≤1	>1/ ≥4	≤ 0.001/ ≤1	>1/ ≥4
Moxifloxacin	≤ 0.25/ ≤0.5	>0.25/ ≥2	≤ 0.25/ ≤0.5	>0.25/ ≥2
Vancomycin	≤ 4/ ≤4	>4/ ≥32	≤ 4/ ≤4	>4/ ≥32
Linezolid	≤ 4/ ≤4	>4/ ≥8	≤ 4/ ≤4	>4/ ≥8
Rifampicin	≤ 0.06/ ≤1	>0.06/ ≥4	≤ 0.06/ ≤1	>0.06/ ≥4
***Enterobacterales (e.g. Escherichia coli***,*** Klebsiella pneumoniae)***				
Ampicillin, amoxicillin	≤ 8/ ≤8	>8/≥ 32	≤ 8/ ≤8	>8/ ≥32
Piperacillin	≤ 8/ ≤8	>8/ ≥32	≤ 8/ ≤8	>8/ ≥32
Piperacillin-tazobactam*	≤ 8/ ≤8	>8/ ≥32	≤ 8/ ≤8	>8/ ≥32
**Cefotaxime, ceftriaxone**	**≤1/** ≤1	**>2/** ≥4	**≤1/** ≤1	**>1/** ≥4
**Meropenem**	**≤2/** ≤1	**>8/** ≥4	**≤2/** ≤1	**>2/** ≥4
Meropenem-vaborbactam*	≤ 8/ ≤4	>8/ ≥16	≤ 8/ ≤4	>8/ ≥16
**Ciprofloxacin**	≤ **0.25/** ≤0.25	**>0.5/** ≥1	**≤0.125/** ≤0.25	**>0.125/** ≥1
Levofloxacin	≤ 0.5/ ≤0.5	>1/ ≥2	≤ 0.5/ ≤0.5	>1/ ≥2
Moxifloxacin	≤ 0.25/--	>0.25/--	≤ 0.25/--	>0.25/--
***Pseudomonas aeruginosa***				
Piperacillin	≤ 0.001/ ≤16	>16/ ≥64	≤ 0.001/ ≤16	>16/ ≥64
Piperacillin-tazobactam*	≤ 0.001/ ≤16	>16/ ≥64	≤ 0.001/ ≤16	>16/ ≥64
Ceftazidime, cefepime	≤ 0.001/ ≤8	>8/ ≥32	≤ 0.001/ ≤8	>8/ ≥32
Ceftazidime-avibactam*	≤ 8/ ≤8	>8/ ≥16	≤ 8/ ≤8	>8/ ≥16
**Meropenem**	**≤2/** ≤2	**>8/** ≥8	**≤2/** ≤2	**>2/** ≥8
Meropenem-vaborbactam*	≤ 8/--	>8/--	≤ 8/--	>8/--
Ciprofloxacin	≤ 0.001/ ≤0.5	>0.5/ ≥2	≤ 0.001/ ≤0.5	>0.5/ ≥2
Levofloxacin	≤ 0.001/ ≤1	>2/ ≥4	≤ 0.001/ ≤1	>2/ ≥4
***Acinetobacter spp.***				
**Meropenem**	**≤2/** ≤2	**>8/** ≥8	**≤2/** ≤2	**>2/** ≥8
Ciprofloxacin	≤ 0.001/ ≤1	>1/ ≥4	≤ 0.001/ ≤1	>1/ ≥4
Levofloxacin	≤ 0.5/ ≤2	>1/ ≥8	≤ 0.5/ ≤2	>1/ ≥8
Colistin	≤ 2/ ≤2	>2/ ≥4	≤ 2/ ≤2	>2/ ≥4
Trimethoprim-sulfamethoxazole	≤ 2/ ≤2^§^	>4/ ≥4^§^	≤ 2/ ≤2^§^	>4/ ≥4^§^

Bold types: special breakpoints for CNS infections available (either EUCAST or CLSI)

#CLSI explicitly states that this is a non-meningitis breakpoint

^§^Trimethoprim-sulfamethoxazole ratio 1:19. Breakpoints are expressed as trimethoprim concentration

IE = insufficient evidence (EUCAST)

*For susceptibility testing, the concentration of clavulanate is fixed at 2 mg/l, of avibactam and tazobactam at 4 mg/l and for vaborbactam at 8 mg/l. The clavulanate and tazobactam in-vitro concentrations are by far above the maximum concentrations reached in the CSF with approved dosing regimens: clavulanate after a single dose of 200 mg 0.28 ± 0.21 mg/l (AUC_CSF_/AUC_plasma_ 0.084) with moderately or severely inflamed meninges [14] and 0.066 ± 0.035 mg/l (AUC_CSF_/AUC_serum_ 0.037 ± 0.015) with uninflamed meninges (Sörgel, in [7]; online supplement); tazobactam after a single dose of 500 mg 0.50 ± 0.40 mg/l (AUC_CSF_/AUC_serum_ 0.170 ± 0.232) with uninflamed or mildly inflamed meninges [15], tazobactam after a single dose of 1 g all except one measured CSF tazobactam concentrations below 4 mg/l [16]. Conversely, in one patient with severe meningeal inflammation treated with avibactam 3 × 500 mg/d, avibactam CSF concentrations of about 4 mg/l were reached [17]. In a ventriculitis patient receiving vaborbactam 3 × 2 g/d, CSF concentrations of vaborbactam were approx. 3-6 mg/l [18]

## Pharmacodynamics of antibiotics in the CNS

Bacterial killing in CSF often is slower than in many extracerebral compartments, because the activity of many antibiotics depends on the speed of replication of the causative pathogens [19–21]. Since CSF is a nutrient-deficient medium, pathogens in the CNS usually replicate slower than in blood or broth. The pH of the CSF in CNS infections often is lower than in other body fluids. Since some patients are experiencing CNS infections with bacteria resistant to all established antibiotics by standard MIC testing, it has been suggested to perform antibiotic testing not only in Mueller-Hinton broth or another classical bacteriologic medium designed for optimum bacterial growth, but in a medium designed to better resemble the chemical constitution of normal human body fluids [22, 23]. Indeed, regarding individual strains, the MICs and MBCs of some antibiotics (e.g. gentamicin, levofloxacin and linezolid for staphylococci, vancomycin for staphylococci and *Streptococcus pneumoniae*, amikacin for *Acinetobacter baumannii*, cefotaxime, ceftazidime and amikacin for *Morganella morganii*, ciprofloxacin and ceftazidime for *Pseudomonas aeruginosa*) were lower in human CSF than in standard culture media [24, 25]. The lowering of the MIC and MBC in CSF did not exceed two titers compared to standard broth (Mueller-Hinton broth/ Brain-heart infusion).

Limited data suggest that - as in extracerebral infections [26]- β-lactam agents, fosfomycin, linezolid and vancomycin in the CNS act as time-dependent antibiotics, whereas fluoroquinolones and aminoglycosides act as concentration-dependent antibiotics [20]. Because of the delayed drug entry and elimination in the CNS, the pharmacokinetic peculiarities of the CNS compartments blur the differences between time-dependent and concentration-dependent antibiotics. In experimental meningitis, antibiotics reach maximum activity at CSF concentrations 10–30 times above the MBC (for β-lactam antibiotics usually identical to or one titer above the MIC) [19, 20]. Slow bacterial killing is achieved at CSF concentrations close to or slightly above the MIC [19].

## Definition of minimal inhibitory concentration (MIC) breakpoints

The concept of relating antibiotic concentrations in vivo to the MIC or MBC determined in vitro has been questioned as a consequence of inter-laboratory and inter-day variations of MIC/MBC determination [21]. The exact determination of a MIC “requires a number of replicate measurements and not a single measurement” [21]. The difficulties in determining exact MICs/MBCs led to the concept of the epidemiological cut-off value (ECOFF): the ECOFF is defined as the upper end of the MIC distribution in wild-type isolates devoid of phenotypically detectable resistance [21]. ECOFFs have been considered for the definition of breakpoints: “In many situations, the ECOFF is similar to the clinical breakpoint” [21].

Breakpoints often, but not always differ according to the site of infection. For some (but not all) of the bacteria causing CNS infections and of the antibiotics used to treat these infections, special breakpoints have been defined (see Table 1). The 2025 CLSI list [5] contains fewer specific breakpoints for CNS infections than the EUCAST list [6]. For several antibiotics used for nosocomial CNS infection, special breakpoints for meningitis/ventriculitis are lacking. In these conditions, the breakpoints valid also for CNS infection refer to the free plasma concentrations during intravenous dosing, which frequently are much higher than the CSF concentrations during intravenous therapy. Whether special breakpoints for CNS infection have been defined or not, sometimes appears arbitrarily (e.g. ciprofloxacin versus levofloxacin and moxifloxacin for *Enterobacterales*). No separate breakpoints have been defined for intrathecal antibiotic regimens [5, 6], where high CSF concentrations are obtained [12, 13].

With respect to β-lactam/β-lactamase inhibitor combinations, standard MIC testing is performed in the presence of fixed β-lactamase inhibitor concentrations [4–6] (Table 1). In the current CLSI and EUCAST protocols, fixed β-lactamase inhibitor concentrations are based on therapeutically relevant free plasma levels to ensure test standardization and consistent inhibitory activity. While this concept is appropriate for bloodstream infections and infections in many other compartments, the unique pharmacokinetic conditions in the CNS require a different approach. 

The β-lactamase inhibitor concentrations used for routine MIC/MBC testing are higher than those usually reached in CSF with approved dosing regimens. For this reason, the MIC testing as performed presently in microbiology routine laboratories must be interpreted with great caution for the assessment of the susceptibility of CNS pathogens to β-lactam/β-lactamase inhibitor combinations. MICs and MBCs of β-lactam/β-lactamase inhibitor combinations of pathogens causing CNS infections should be tested in vitro in the presence of β-lactamase inhibitor concentrations achievable in human CSF by regular doses. One provisional solution could be to test β-lactam/β-lactamase inhibitor combinations at the concentration ratio administered in commercially available drugs over the whole range of dilutions for MIC/MBC testing, i.e., diluting both the β-lactam antibiotic and the β-lactamase inhibitor instead of only diluting the β-lactam. Since the molecular weight (MW) of the β-lactamase inhibitor is smaller than the MW of the β-lactam, the penetration into CSF of the β-lactamase inhibitor usually is slightly greater than the CSF penetration of the respective β-lactam [7, 14, 15]. Diluting both the β-lactam and the β-lactamase inhibitor for MIC/MBC testing (as it is routinely performed for both components of trimethoprim-sulfamethoxazole) would not entail the risk of overestimating the activity of a β-lactam/ β-lactamase inhibitor combination in CSF, but would imply a small risk of underestimating the activity of a β-lactam/ β-lactamase inhibitor combination in CSF due to the slightly higher penetration of the β-lactamase inhibitor into the CSF. Since CSF pharmacokinetics vary widely with inflammation, CSF flow, renal function, and other clinical variables, additional pharmacokinetic/ pharmacodynamic studies are necessary to define physiologically relevant β-lactamase inhibitor concentrations for susceptibility testing in CNS infections.

## Pharmacokinetic/ pharmacodynamic considerations

In view of the pharmacokinetics and pharmacodynamics of antibiotics in the CSF, for a clinician using ceftriaxone or cefotaxime to treat *Escherichia coli* meningitis in an infant or an elderly patient, the information whether the MIC of the causative pathogen is 0.03 µg/ml (highly susceptible) or 1 µg/ml (according to CLSI or EUCAST criteria still considered fully susceptible) is of great importance. Although ceftriaxone and cefotaxime are time-dependent antibiotics, in experimental rabbits bacterial killing under the special conditions of the CSF space was more rapid at antibiotic CSF concentrations ≥ 10x MBC than at antibiotic CSF concentrations close to the MBC [19]. For most bacteria exposed to β-lactam antibiotics, the MBC is equal or 1–2 titers higher than the MIC. Assuming a ceftriaxone or cefotaxime steady state concentration in the ventricular CSF of approx. 1 mg/l (a realistic value in the absence of strong meningeal inflammation) [7], for the highly susceptible *E. coli* (MIC 0.03 µg/ml) a C_CSF_/MIC ratio of 30 can be expected ensuring a large safety distance between C_CSF_ and MIC and rapid killing. Conversely, for an *E. coli* with a MIC of 1 µg/ml still considered fully susceptible, the MIC is close to the ventricular cephalosporine CSF concentration making a rapid in vivo bactericidal effect unlikely. Maintaining antibiotic CSF concentrations relative to the MIC is crucial not only during the initial inflammatory phase but also during the later treatment phase when permeability of the blood-CSF barrier decreases. The knowledge of the exact MIC and of the CSF concentrations of the antibiotic in the absence of meningeal inflammation enable the clinician to estimate, whether the antibiotic CSF concentrations during resolution of inflammation are high enough to prevent treatment failure.

For this reason, in CNS infection, we believe that to give up the exact determination of MICs is a setback. We suggest the reporting of exact MICs to the clinician in addition to classifying bacteria as “S”, “I” or “R” according to breakpoints. The lack of the determination of exact MICs may be a risk, particularly when no special clinical breakpoints for CNS infections have been defined. As a consequence of the variation occurring during MIC determination [21], it may be prudent to determine MICs in triplicate and report the median in infections with highly resistant bacteria and cases where it is difficult to achieve pharmacokinetic/ pharmacodynamic targets. Moreover, in the presence of highly resistant bacteria, where MIC determination in broth does not offer a treatment option (e.g. in ventriculitis caused by extensively drug-resistant Acinetobacter spp.), MIC and MBC testing in human CSF instead of broth may be appropriate to identify antibiotics, whose CSF levels exceed the MIC determined in CSF in vitro and which can be expected to have antibacterial activity in vivo. Due to the limited CSF sample volume, variable matrix quality, and lack of standardized methodology, further research should focus on the development of defined artificial CSF media with controlled composition and pH. For CNS infections, β-lactam/β-lactamase inhibitor combinations should be tested with β-lactamase inhibitor concentrations achievable in CSF with regular doses.

## Data Availability

No datasets were generated or analysed during the current study.
